# Leaf extracts from *Nitraria retusa *promote cell population growth of human cancer cells by inducing apoptosis

**DOI:** 10.1186/1475-2867-11-37

**Published:** 2011-10-31

**Authors:** Jihed Boubaker, Wissem Bhouri, Mohamed Ben Sghaier, Ines Bouhlel, Ines Skandrani, Kamel Ghedira, Leila Chekir-Ghedira

**Affiliations:** 1 Laboratory of Cellular and Molecular Biology, Faculty of Dental Medicine, University of Monastir, Rue Avicenne, Monastir, 5000, Tunisia; 2 Unity of Pharmacognosy/Molecular Biology, Faculty of Pharmacy, University of Monastir, Rue Avicenne, Monastir, 5000, Tunisia

## Abstract

**Background:**

In this report the phytochemical profile of *Nitraria. Retusa (N. Retusa*) leaf extracts were identified and their ability to induce apoptosis in human chronic myelogenous erythroleukaemia (K562) was evaluated.

**Methods:**

Apoptosis of the human chronic myelogenous erythroleukaemia (K562) was evidenced by investigating DNA fragmentation, PARP cleavage and caspases 3 and 8 inducing activities, in the presence of *N. retusa *extracts.

**Results:**

Our study revealed that the tested extracts from *N. Retusa *contain many useful bioactive compounds. They induced in a time-dependent manner the apoptosis the tested cancerous our cell line. This result was confirmed by ladder DNA fragmentation profile and PARP cleavage, as well as a release in caspase-3 and caspase-8 level.

**Conclusion:**

Our results indicate that the tested compounds have a significant antiproliferative effect which may be due to their involvement in the induction of the extrinsic apoptosic pathway.

## Background

Apoptosis is a form of cell death in which a programmed sequence of events eliminates cells without damaging neighbouring cells. Apoptosis is triggered through either a death receptor mediated extrinsic pathway or a mitochondrial intrinsic pathway. Phytotherapy is considered as an alternative, to mitigate side effects due the indiscriminate use of synthetic drugs. For many years, the antiproliferative actions of chemotherapeutic drugs were ascribed solely to their ability to induce genotoxic damage [1]. Therefore, the role of plant derived polyphenols in chemoprevention of cancer has emerged as an interesting area of research. To date, many anticancer drugs have been developed and applied by clinical doctors [2]. In addition flavonoids have been shown to cause apoptosis through induction of Bax with concomitant suppression of Bcl-2, or through other molecules and pathways including up-regulation of death receptor 5, modulation of IGFBP-3, involvement of p38-MAPK, and inhibition of PI-3-kinase/Akt and ERK pathways [3]. In our case, we were interested with leaf extracts from *Nitraria retusa *in order to investigate an alternative phytoterapy solution to current anticancerous treatments. Its fleshy red fruits are eaten by humans and are used to prepare drinks. The leaves serve as supplement for the tea and are used as poultice [4]. The ashes of this species have the ability to remove fluids of infected wounds [5]. Belkadhar [6] indicates that a decoction of fresh leaves of *Nitraria retusa *is used in Morocco in case of poisoning, upset stomach, ulcers, gastritis, enteritis, heartburn, colitis, colonic abdominal pain. In this study, we analyzed and compared cytotoxic effects of hexane, chloroform and methanol extracts, on a human chronic myelogenous erythroleukaemia (K562) cell line. We attempt to elucidate the apoptotic pathway and molecular mechanisms responsible for their cytotoxic and apoptotic activities.

## Methods

### Reagents

All the organic solvents were obtained from Carlo ERBA (Paris, France). L-glutamine was purchased from GIBCO BRL Life technologies (Grand Island, NY, USA). The chromatographic columns were performed with silica gel 60 (Pharmacia Biotech, Uppsala, Sweden), reverse phase C18 column (Merck, Darmstadt, Hesse, Germany). The *N*-(1-naphtyl) ethlenediaminedihydrochloride (EDTA) was purchased from Sigma-Aldrich (Steinheim, Germany). Dimethylsulfoxide (DMSO), monoclonal antibody *i.e *anti poly ADP-ribose polymerase (anti-PARP), goat anti mouse alkaline phosphtase conjugated antibody, caspase-3 and caspase-8 colorimetric assay kits and 3-(4, 5-dimethylthiazol-2-yl)-2, 5-diphenyl tetrazolium) (MTT) were purchased from Sigma RBI, (St.Louis, MO, USA). RPMI-1640, foetal bovine serum and gentamicin were bought from GIBCO BRL Life technologies (Grand Island, NY, USA). The proteinase K, the sodium dodecyl sulfate (SDS), ribonuclease (RNase), Sarkosyl, Thiobarbituric Acid (TBA), and pyridine were purchased from Sigma Aldrich Co (St. Louis, MO, USA). Acrylamide and bisacrylamide, 5-bromo-4 chloro-3 indolyl phosphate (BCIP)/nitro blue tetrazolium (NBT) and tween 20, were purchased from promega (Madison, Wisconsin, USA). Ethidium bromide (EtBr) and bromophenol blue were purchased from Merck (Darmstadt, Hesse, Germany). Agarose and ployvinylidene difluoride (PVDF) membranes were obtained from Invitrogen, life technologies (Glasgow, UK). Acetic acid was procured from Panreac (Barcelone, Espagne).

### Plant Material

Leaves of *N. retusa *were collected from saline soils in Sahline, a region situated in mid-Tunisia, in December 2006. Identification was carried out by Pr. M. Cheieb (Department of Botany, Faculty of Sciences, University of Sfax, Sfax, Tunisia), according to the Flora of Tunisia [7] and Contribution to ethnobotanical study of the flora of Tunisia [8]. A voucher specimen (N.r-12.06) was kept in our laboratory for future reference. The leaves were hade dried, powdered, and stored in a tightly closed container for further use.

### Preparation of plant extracts

Three hundred and fifty grams of powder, from dried leaves, were sequentially extracted in a Soxhlet apparatus (6 h) (AM Glassware, Aberdeen, Scotland, United Kingdom) with hexane, chloroform, ethyl acetate and methanol solvents. We obtained the correspondent extracts for each solvent. Hexane (Hex), chloroform (Chl) and methanol (MeOH) extracts, with different polarities, were concentrated to dryness and the residues were kept at 4°C. Then, each extract was resuspended in dimethyl sulfoxide solvent (DMSO).

### Preliminary phytochemical analysis and determination of Total Polyphenol, Flavonoid, Tannins and Sterol Contents

Plant materials were screened for the presence of tannins, flavonoids, coumarins and sterols using the methods previously described by Tona *et al. *[9,10].

The polyphenol content of *N. retusa leaf *extracts was quantified by the Folin-Ciocalteau reagent as described by Yuan *et al. *[11]. The Gallic acid (0.2 mg/ml) was used as a standard. The polyphenol content was expressed according to the following formula:

$$\%\text{ Polyphenols}=\left(\left[{\text{A}}_{\left(\text{72}0\text{nm}\right)}\text{extract}\times 0.\text{2}\right)/{\text{A}}_{\left(\text{72}0\text{nm}\right)}\text{Quercetin}\right]/\text{Extract concentration})\times 100$$

However, flavonoid content was determined according to the modified method of Zhishen *et al. *[12]. The Quercetin (0.05 mg/ml) was used as a standard compound.

The flavonoïd content was expressed according to the following formula:

$$\%\text{ Flavonoids}=\left(\left[{\text{A}}_{\left(\text{51}0\text{nm}\right)}\text{extract}\times 0.0\text{5}\right)/{\text{A}}_{\left(\text{51}0\text{nm}\right)}\text{Gallic acid}\right]/\text{ Extract concentration})\times 100$$

The total sterol content was evaluated as described by Skandrani *et al. *[13]. The sterol content was expressed according to the following formula:

$$\%\text{Sterols = }\left(\text{W steroids}∕\text{W extract}\right)\times 100.\text{ Where Wsteroids = }\left(\text{Wf}-\text{WO}\right)\times 0.\text{25}$$

MO: Weight filter (mg), Mf: Weight of filter with the precipitate (mg).

The method described by Pearson [14], was used for the determination of tannin content of samples which is evaluated according to the following formula:

$$\%\text{ Tanins}=([{\text{A}}_{\left(\text{76}0\text{nm}\right)}\text{extract }/.\;\text{ε}\times \text{1})/\text{ Extract concentration}\times 100$$

where ε; molar extinction coefficient (= l g-1 cm-1) of tannic acid (= 3.27 l g-1 cm-1)

### Cell cultutre

Human chronic myelogenous leukemia cell line K562 was obtained from the American Type Culture Collection (Rockville, MD). Cells were cultivated in RPMI-1640 medium supplemented with 10% (v/v) foetal calf serum, 0.1 mg/ml gentamicin and 2 mM L-glutamine as a complete growth medium and were incubated at 37)°C in an incubator with 5% CO_2 _in a humidified atmosphere. Every two days the cells were subcultured by splitting the culture with fresh medium.

### Assay for cytotoxic activity

Cytotoxicity of *Nitraria retusa *extracts against K562 leukemia cells was estimated by the 3-(4, 5-dimethylthiazol-2-yl)-2, 5-diphenyltetrazolium bromide (MTT) assay, based on the reduction of the MTT by mitochondrial dehydrogenases in viable cells. The resulting blue formazan product is measured spectrophotometrically [15]. Cells were seeded in a 96-well plate at a concentration of 5 × 10^4 ^cells/well and incubated at 37°C for 24 h in a 5% CO_2 _enriched atmosphere. The extracts were firstly dissolved in 1% DMSO, then in the cell growth medium. Cells were incubated again at 37°C for 48 h with each of the tested extract at concentrations ranging from 10 to 800 μg/ml. Next, the medium was removed and cells in each well were incubated with 50 μl of MTT solution (5 mg/ml) at 37°C for 4 h. MTT solution was then discarded and 50 μl of 100% DMSO were added to dissolve the insoluble formazan crystal. The optical density was measured at 540 nm. Each drug concentration was tested in triplicate.

The cytotoxic effects of the extracts were estimated in terms of cell population growth inhibition percentage and expressed as IC_50 _which is the concentration of extract that reduces the absorbance of the treated cells by 50% with reference to the control (cells treated with DMSO). The IC_50 _values were graphically obtained from the dose-response curves. We determined IC_50 _values when cytotoxicity resulted more than 50% at screening concentrations.

### Evaluation of lipid peroxidation induction provoked by H_2_O_2_, using the thiobarbituric acid reactive substances (TBARS) assay

The method known as thiobarbituric acid reactive species (TBARS) assay, concerns the spectrophotometric measurement of the pink pigment produced through reaction of thiobarbituric acid (TBA) with malondialdehyde (MDA) and other secondary lipid peroxidation products. TBARS were determined by previously described assay [16]. The cells (3.5 10^7 ^cells/ml) were exposed to various concentrations of each compounds (200, 400 and 800 μg/ml of MeOH extract, 150, 300 and 600 μg/ml of hexane extract and 190, 380 and 760 μg/ml of chloroform extract) in the incubation medium during 2 h, followed by incubation with 75 mM _H2O2 _for 2 h. The ranges of doses of different tested compounds were chosen on basis of their cytotoxic activity. The cells were washed with PBS, pelleted and homogenized in 1.15% KCl. Samples were combined with 0.2 ml of 8.1% SDS, 1.5 ml of 20% acetic acid and 1.5 ml of 0.8% thiobarbituric acid. The mixture was brought to a final volume of 4.0 ml with distilled water and heated to 95°C for 120 min. After cooling for 10 min on ice, 5.0 ml of a mixture of n-butanol and pyridine (15:1 v/v) were added to each sample, and the mixture was shaken vigorously. After centrifugation at 825 g for 10 min, the supernatant fraction was isolated and the absorbance was measured at 532 nm. Lipid peroxidation effect was expressed as equivalent of MDA. Data were reported as mean ± SD for triplicate determinations.

### DNA fragmentation analysis

DNA fragmentation was analysed by agarose gel electrophoresis as described by Wang *et al *[17], with slight modifications. K562 cells (1.5 106 cells/ml) were exposed to various concentrations of each compounds (200, 400 and 800 μg/ml of MeOH extract, 150, 300 and 600 μg/ml of hexane extract and 190, 380 and 760 μg/ml of chloroform extract) for 24 and 48 h and harvested by centrifugation. Cell pellets were disolvedin 200 μl of lysis buffer (50 mM Tris-HCl, pH 8.0, 10 mM EDTA, 0.5% N-Lauroyl Sarcosine Sodium Salt) at room temperature for 1 h then centrifuged at 12 000 g for 20 min at 4°C. The supernatant was incubated overnight at 56°C with 250 μg/ml proteinase K. Cell lysates were then treated with 2 mg/ml RNase A and incubated at 56°C for 2 h. DNA was extracted with chloroform/phenol/isoamyl alcohol (24/25/1, v/v/v) and precipitated from the aqueous phase by centrifugation at 14 000 g for 30 min at 0°C. The DNA solution was transferred to 1.5% agarose gel and electrophoresis was carried out at 67 V for 3/4 h with TAE (Tris 40 mM, sodium acetate 20 mM, EDTA 1 mM) as the running buffer. DNA in the gel was visualized with ethidium bromide (0.5 μg/ml) under UV light.

### Western blot analysis

K562 cells (1.5 10^6 ^cells/ml) were exposed to various concentrations of each compounds (200, 400 and 800 μg/ml of MeOH extract, 150, 300 and 600 μg/ml of hexane extract and 190, 380 and 760 μg/ml of chloroform extract) for 6, 24 and 48 h. Cells were centrifuged at 3000 rpm for 8 min at 25°C and lysed with a lysis buffer (62.5 mM Tris Hcl and 6 mM urea, pH = 6.8). Protein concentrations were determined in cell lysates using the Bradford method [18]. Equal amounts of proteins (40 mg) were separated by sodium dodecyl sulphate polyacrylamide gel electrophoresis (SDS-PAGE), and transferred into PVDF membrane, which was blocked with 5% non-fat milk in 0.1% Tween 20-phosphate buffer salin (PBST) overnight at 4°C. Membranes were then incubated with a primary antibody anti-PARP at a 1:100 dilution for 2 h at room temperature. The membrane was then washed and incubated with a goat anti-mouse alkaline phosphatase-conjugated antibody at 1:7500 dilution for 1 h.

Next, the membrane was washed and the chromogenic substrate BCIP/NBT was added to localise antibody binding proteins. Protein levels were determined by computer assisted densitometric analysis (Densitometer, GS-800, Bio.Rad Quantity One).

### Investigation of caspase-3 and caspase-8 induction

The cells were cultured (10^6 ^cells/ml) in 25 cm^2 ^flasks for 24 h in the absence or the presence of extracts at 37°C. Controls were performed at the same time with 0, 5% DMSO. The cells were harvested and centrifuged at 600 × *g *and the pellets were incubated in ice cold lysis buffer (250 mM HEPES, pH 7.4, 25 mM CHAPS, 25 mM DTT) for 15 min, then they were centrifuged at 16000 × *g *for 20 min. Supernatants (cell extracts eventually containing caspase-3 and caspase-8) were retrieved and aliquots corresponding to 50 μg total protein were incubated along with acetylated tetrapeptide Ac-DEVD substrate labelled with the chromophore *p*-nitroaniline (*p-*NA), 2 mM, for caspase 3 assay, or Ac-IETD-*p*-NA substrate, 2 mM, for caspase 8 assay, in the presence of each caspase buffer in a 96-well flat bottomed microplate.

In the presence of active caspase-3 and caspase-8, cleavage and release of *p*-NA from the substrate occurs. Free *p-*NA produces a yellow colour detected spectrophotometrically at 405 nm against a blank performed at the same time and containing assay buffer (200 mM HEPES, pH 7.4, 1% CHAPS, 50 mM DTT, 20 mM EDTA, for caspase 3 assay) and (200 mM HEPES, pH 7.4, 1% CHAPS, 50 mM DTT, 20 mM EDTA, 50% sucrose for caspase 8 assay) and substrate but without cell lysate. A standard curve was performed in order to determine the correspondence between absorbance and *p-*NA concentration, then the results were expressed as caspase-3 and caspase-8 specific activity (μmol *p*-NA per min/ml protein) calculated as indicated by the manufacturer (Caspase-3, caspase-8, assay kit colorimetric, Sigma.)

### Statistical analysis

Data were collected and expressed as the mean ± standard deviation of three independent experiments and analyzed for statistical significance from control. The data were tested for statistical differences by one-way ANOVA followed by student test using statistica. The criterion for significance was set at *p *< 0.05.

## Results

### Phytochemical study and determination of extract yield, total polyphenol, flavonoid, tannin and sterol contents of *Nitraria retusa *leaf extracts

Using 350 g of powder from the leaves of *N retusa*, we obtained 50 g of MeOH extract, 10 g of Hex extract and 6 g of Chl extract, corresponding to yields of 14.30%, 3.50%, and 1.71% respectively (Table 1).

**Table 1:** Phytochemical screening of extracts from Nitraria retusa:

	Hex extract	Chl extract	MeOH extract
Sterols	++++	++	-
Flavonoids	-	-	+++
Tanins	-	-	+++
Coumarins	-	++	-
polyphenols	-	++	+
Yield (%)	3.5	1.71	14.3

- = not detectable; + = low quantitiy; ++ = average quantitiy +++ = high quantitiy ++++ = very high quantitiy

The highest content of polyphenols was recorded in Chl extract. In fact, the percentage of the total polyphenolic content in chloroform extract was 10.03%. The Hex extract showed the presence of an important quantity of sterols equivalent to 31%. Whereas, MeOH extract exhibited the highest quantities of tannins and flavonoids. The percentages of tannins and flavonoids in MeOH extract were respectively 18.84% and 14.82% (Table 2).

**Table 2:** Quantitative phytochemical screening of extracts from Nitraria retusa leaves

Extract content (%)	Hex extract	Chl extract extract	MeOH extract
Tanins(%)	-	-	18.84 ± 0.002
Flavonoid(%)	-	-	14.82 ± 0.009
Polyphenols (%)	-	10.03 ± 0.008	3.3 ± 0.006
sterols(%)	31 ± 0.02	11.75 ± 0.007	-

(results are represented by the means ± SD of three experiments)

### Evaluation of the cytotoxicity of extracts

**Figure 1:**
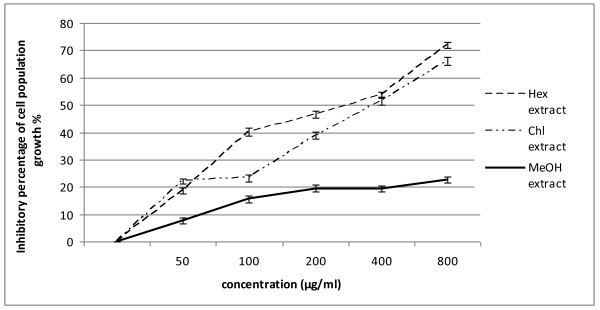
**Inhibitory effect, of *Nitraria retusa *extracts on the viability of K562 cells**. Results are represented by the means ± SD of n = 3. (*) p < 0.05 means a significant difference between the untreated and treated cells. Hex: hexane extract, Chl: chloroform extract, MeOH: methanol extract.

We have examined the effect of different concentrations (200, 400 and 800 μg/ml) of each extract on the K562 cell population growth *in-vitro*, using the MTT assay. The results of this assay are reported in Figure 1. Hex and Chl extracts inhibited strongly the malignant tested cell population growth (IC_50 _values were 300 and 380 μg/ml respectively). However, no significant cytotoxic activity was revealed in the presence of MeOH extract (IC_50 _> 800 μg/ml).

### Effect of extracts on lipid peroxidation induced by H_2_O_2_

The reaction of MDA with TBA has been widely adopted as a sensitive assay method for lipid peroxidation. The effect of different concentrations of *Nitraria retusa *extracts on malondialdehyde (MDA) production in K562 cells, induced by H_2_O_2_, is shown in (Table 3). Hexane and chloroform extracts showed a protective effect against lipid peroxidation induced by H_2_O_2 _at the highest tested concentrations. The lipid peroxidation effect evaluated as MDA equivalent produced determined by using the TBARS test, were 150, and 190 nM at a concentration of 600 μg/ml of Hex extract and 760 μg/ml of Chl extract. All the other tested concentrations for each extract showed an amplifying action of H_2_O_2 _pro-oxidant effect. In fact the obtained values were higher than those showed with H_2_O_2 _only (225 nM). While, MeOH extract showed a H_2_O_2 _pro-oxidant amplifying effect at all the tested concentrations, whereas alone it did not exhibit any inductive effect of lipid peroxidation (data not shown).

**Table 3:** Lipid peroxidation inhibitory activity in K562 cells treated with Hex, Chl and MeOH extracts against H_2_O_2 _(50 μM) induced peroxidation.

extraits	Concentration (μg/ml) ^a^	Concentration of MDA (nM)
**Hex**	150	260 ± 16
	
	300	230 ± 11
	
	600	150 ± 10

**Chl**	190	245 ± 15
	
	380	220 ± 14
	
	760	190 ± 20

**MeOH**	200	250 ± 10
	
	400	265 ± 5
	
	800	315 ± 10

**H_2_O_2_**	50 μM	225 ± 5

^a ^results are represented by the means ± SD of three experiments

(*) *p *< 0.05 means a significant difference between the treated cells with different extracts and treated cells with hydrogen peroxide (H_2_O_2_).

### Induction of apoptotic DNA fragmentation by *Nitraria retusa *extracts on leukemia cells

**Figure 2:**
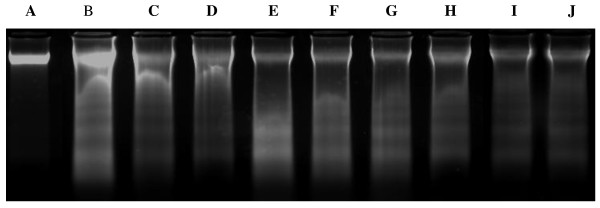
**DNA electrophoretic profiles of k562 cells treated with different concentrations of Hex (hexane), Chl (chloroform) and MeOH (methanol) extracts during 48 h h**. DNA was separated on 1.5% agarose gel. **A: **PC: K562 cell DNA; DNA of cells treated with **B: **Hex 600 μg/assay Hex extract, **C: **300 μg/assay Hex extract, **D**: 150 μg/assay Hex extract, **E**: 780 μg/assay Chl extract, **F**: 380 μg/assay Chl extract, **G**: 190 μg/assay Chl extract, **H**: 200 μg/assay MeOH extract, **I**: 400 μg/assay MeOH extract, **J: **800 μg/assay MeOH extract.

At exposure with different concentrations of Hex extract (Figure 2, tracks B, C, D), Chl extract (Figure 2, tracks E, F, G) and MeOH extract (Figure 2, tracks H, I, J) during 48 h, a fragmented DNA profile was clearly observed in K562 cells, compared to untreated cells which did not provide a ladder DNA profile (Figure 2, track A).

### Effect of *Nitraria retusa *extracts on the proteolysis of PARP

DNA fragmentation is often associated with the activation of a family of cysteine proteases, the caspases. Caspase-3, in particular, seems to play an important role in several models of apoptosis [19]. To confirm the apoptotic process, generally admitted when a ladder DNA fragmentation profile is observed, we investigated the enzymatic activation of apoptotic proteins by measuring the cleavage of PARP (116 kDa), which is a caspase-3 substrate, into fragments of 85 and 31 kDa.

**Figure 3:**
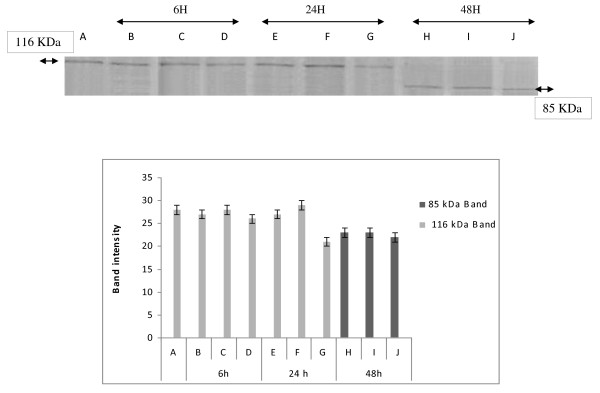
**Changes in expression of apoptosis-related protein in response to treatment with hexane extract and quantification by scanning densitometry of PARP bands intensity**. K562 cells were treated with 600, 300 and 150 μg/ml of hexane extract (Hex) for 6, 24 and 48 h. Protein extracts were subjected to western blotting to determinate immunoreactivity of PARP, as described in methods section. PARP 116 KDA and 85KDA bands are shown. Quantification by scanning densitometry of PARP bands intensity (quantified band intensity by computer-assisted densitometric analysis (Densitometer, GS-800, BioRad Quantity One)). **A**: Untreated cells, **B: 60**0 μg/ml, **C: 30**0 μg/ml, **D**: 150 μg/ml, **E**: 600 μg/ml, **F**: 300 μg/ml, **G**: 150 μg/ml, **H**: 600 μg/ml, **I**: 300 μg/ml, **J: 15**0 μg/ml.

As shown in (Figure 3.A and 4.A), when cells were treated with Hex and Chl extracts, the 116 kDa band disappears after 48 h treatment and the band of a 85 kDa arises. Whereas when incubated with MeOH extract, K562 cells exhibited a non fragmented band pattern after 6 h and 24 h incubation. This band disappears totally after 48 h of incubation. However we observed a decrease of the 116 kDa band intensity, after 24 h incubation, and at the same time a 85 kDa band appears. The intensity of this band was higher in cells treated by extract for 48 h than for 24 h. (Figure 5.A)

**Figure 4:**
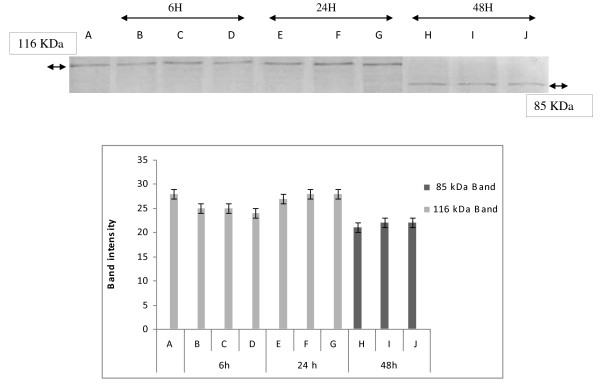
**Changes in expression of apoptosis-related protein in response to treatment chloroform extract and quantification by scanning densitometry of PARP bands intensity**. K562 cells were treated with 760, 380 and 190 μg/ml of chloroform extract for 6, 24 and 48 h. Protein extracts were subjected to western blotting to determinate immunoreactivity of PARP, as described in methods section. PARP 116 KDA and 85 KDA bands are shown. (quantified band intensity by computer-assisted densitometric analysis (Densitometer, GS- 800, BioRad Quantity One)). **A**: Untreated cells, **B: 190 **μg/ml, **C: 38**0 μg/ml, **D**: 760 μg/ml, **E**: 190 μg/ml, **F**: 380 μg/ml, **G**: 780 μg/ml, **H**: 190 μg/ml, **I**: 380 μg/ml, **J: 760 **μg/ml.

**Figure 5:**
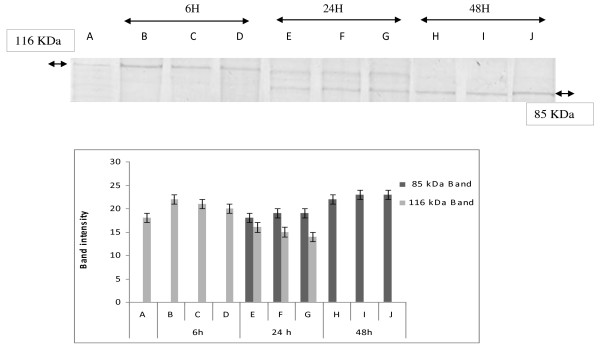
**Changes in expression of apoptosis-related protein in response to treatment methanol extract and quantification by scanning densitometry of PARP bands intensity**. K562 cells were treated with 800, 400 and 200 μg/ml of methanol extract for 6, 24 and 48 h. Protein extracts were subjected to western blotting to determinate immunoreactivity of PARP, as described in methods section. PARP 116 KDA and 85 KDA bands are shown. (quantified band intensity by computer-assisted densitometric analysis (Densitometer, GS-800, BioRad Quantity One)). **A**: Untreated cells, **B: 8**00 μg/ml, **C: 4**00 μg/ml, **D**: 200 μg/ml, **E**: 200 μg/ml, **F**: 400 μg/ml, **G**: 800 μg/ml, **H**: 200 μg/ml, **I**: 400 μg/ml, **J: 80**0 μg/ml.

### Caspase-3 and caspase-8 activation assay

**Figure 6:**
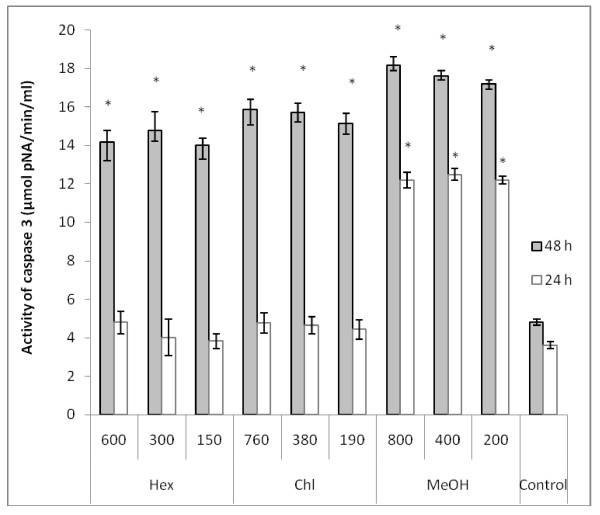
**Effect of Hex, Chl and MeOH extracts on caspase-3 activity in K562 cells**. Lysates prepared from cells treated with *N. retusa *leaf extracts for 24 h and 48 h, were assayed for *in vitro *caspase-3 activity. The rate of cleavage of the caspase substrate DEVD-*p*NA was measured at 405 nm. The results are presented as the mean ± SD. The experiments were done in triplicate. (*) *p *< 0.05 means a significant difference between the control and treated cells Control: cells treated by the vehicle only.

**Figure 7:**
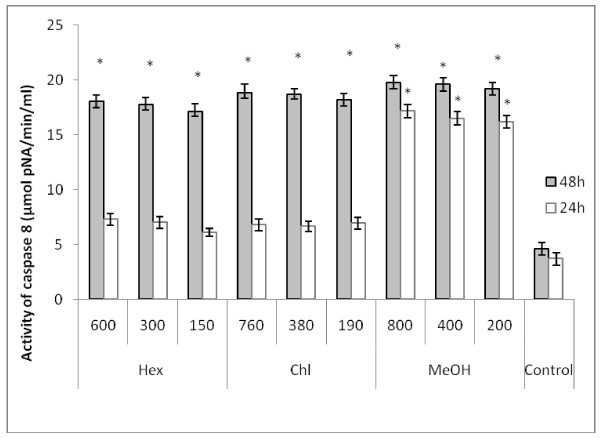
**Effect of Hex, Chl and MeOH extracts on caspase-8 activity in K562 cells**. Lysates obtained from cells treated with *Nitraria retusa *leaf extracts 24 h and 48 h were assayed for *in vitro *caspase-8 activity. The rate of cleavage of the caspase substrate IETD-*p*NA was measured at 405 nm. The results are presented as the mean ± SD. The experiments were done in triplicate. (*) *p *< 0.05 means a significant difference between the control and treated cells Control: cells treated by the vehicle only.

As the proapoptotic PARP is a substrate of caspases, we attempt to investigate the cellular pathway of cell death induced by *Nitraria retusa *extracts, by assessing caspase-3 and caspase-8 activities. These two proteins play a critical role in apoptosis. Following 24 h and 48 h treatment of K562 cells with various concentrations of *Nitraria retusa *extracts, caspase-3 and caspase-8 activities were measured and compared to those of control cells. As shown in Figures 6 and 7, K562 cells treated with *Nitraria retusa *extracts, showed a significant concentration-depending increase of caspase-3 and caspase-8 activities after 48 incubation with Hex and Chl extracts, and after 24 and 48 h inubation with MeOH extract. These results suggest that apoptosis induced by the tested *Nitraria retusa *extracts may occur through the activation of common executors of apoptosis such as caspase-3 by the activation of caspase-8.

## Discussion

The relationship between concentration of extracts and their antiproliferative effect on K562 cells was investigated by MTT assay. Hex, MeOH and Chl extracts possess an inhibitory effect on K562 cell proliferation. The strong antiproliferative activity of Hex may be due to the presence of sterols, which are known to induce antiproliferative effect [20]. In fact, Phytosterols seem to act through multiple mechanisms of action, including inhibition of carcinogen production, cancer-cell growth, angiogenesis, invasion and metastasis, and through the promotion of apoptosis of cancerous cells stress [21]. Inhibition of proliferation of K562 cells exhibited by Chl extract may be attributed to the presence of specific components such as polyphenols [13]. Besides, some studies have shown that polyphenols are able to influence a variety of cell function by modulating cell signalling [22], altering proliferation and induction anti-proliferative effect in cancer cell lines [23]. In fact, polyphenols exhibited antiproliferative effects on various cancerous human cell lines, for example leukaemia cells [24] and ovarian cancer cells [25]. However, minor components could also contribute to the antiproliferative effect of these extracts; they may be involved in some types of synergism with other active compounds [26]. The weak antiproliferative activity exhibited by the MeOH extract should be ascribed to its low polyphenol content fraction, if compared to Hex and Chl extracts. Nonetheless, polyphenols contained in MeOH extract should be different from those Chl extract as far as they were extracted with solvents having different polarities.

Membrane lipids are rich in unsaturated fatty acids that are most susceptible to oxidative processes. It is generally thought that the inhibition of lipid peroxidation by antioxidants may be due to their free radical-scavenging activities. The data obtained showed that Hex and Chl extracts exhibited better antioxidant activity at the highest tested concentrations than MeOH extract. We can deduce that sterols which are the main constituents of Hex extract, and sterol and polyphenolic compounds which are the main constituents of Chl extract, should participate in the protective effect, of these two extracts at the highest tested concentration, against lipid peroxidation induced by H_2_O_2 _in K562 cells. This protective effect is absent at the lowest tested concentrations of the above mentioned extracts. In fact the lowest tested concentrations of both extracts did not exhibit a protective effect against lipid peroxidation induced by H_2_O_2_, as far as the rates of MDA formation obtained when cells are incubated with both H_2_O_2 _and each of these concentrations are in the same range as those obtained when cells are incubated with H_2_O_2 _alone. These results are in accordance with those of Mahoney and Graf [27] who showed that ascorbic acid initiates the formation of OH^• ^at low concentrations and scavenges radicals at high concentrations. It is possible that these compounds inhibit the free radicals and ROS produced by oxidation and redox-cycling started by H_2_O_2 _and leading to cell lipid peroxidation. Although MeOH extract contains flavonoids, tannins and polyphenols, it exhibits no protective effect against lipid peroxidation induces by H_2_O_2_. We believe that its weak polyphenol content and absence of sterols may explain the absence of protective effect against lipid peroxidation. In fact, polyphenols are an important group of pharmacologically active compounds, they are considered to be the most active antioxidant derivatives in plants. However, it has been shown that the phenolic content does not necessarily follow the antioxidant activity. Antioxidant activity is generally the result of the combined activity of a wide range of compounds, including phenolics, peptides, organic acids and other components [28].

However some flavonoids and polyphenols as catechol or pyrogallol may exhibit a prooxidant activity by generating free radicals, under certain conditions [29].

Our hypothesis that sterol contents of both Chl and Hex extracts are involved in their antioxidant effect is in accordance with the results described by Wang *et al. *[30], who reported the antioxidant capacity of some plant sterols. In fact, it is possible that sterols inhibit free radicals and ROS produced by oxidation and redox-cycling, as reported by Argolo et al. [31] and Ben Mansour et al. [32].

An oxidative stress probably provoked by extracts would lead to the formation of free radicals being able to induce cellular stress, as cell DNA degradation and membrane permeabilization especially mitochondrial membranes, provoking liberation of pro-apoptotic proteins as pro-caspases, cytochrome C, which interact with proteins as Apaf-1 and pro-caspase-9 forming, in the presence of ATP, a multi-protein complex named "apoptosome". This complex should allow the cleavage of caspase-9. This later will activated executive caspases as capases-3, 7 and 8, involved in the induction of apoptotic process. On the other hand, cell stress induced by free radicals should also activate a pro-apoptotic gene family (Bax, Bak, Bid, Bad, Bim) or inactivate anti-apoptotic genes as Bcl-2, Bcl-xL [33], inducing thus cell apoptotic process.

The antiproliferative activity of MeOH extract from *N. retusa *should be attributed to the presence of specific types of flavonoids and polyphenols [13]. In fact, some studies have shown that flavonoids [23] and tannins [34] are able to altering proliferation in cancer cell lines. Previous studies have shown that flavonoids induce apoptosis of various tumor cells including K562. This effect has also been observed in other tumor cell lines from gastric, colon and lung carcinomas [35]. In addition, flavonoids also inhibited tumor growth through cell cycle arrest and induced apoptosis through a p53-dependent mechanism [36].

Although *N. retusa *extracts should contain some antioxidant entities (revealed by antiradical properties of the same *N. retusa *extracts against several free radicals; data not shown), we believe that this dual property, reported also in other works [37] is not in contradiction with our aforementioned deduction, as several researchers have shown that antioxidants, such as retinoids and vitamin E, produce genetic changes that cause apoptosis in cancer cells by mechanisms other than a direct antioxidant effect [38].

The typical DNA fragmentation pattern which is considered as the hallmark of apoptosis, was observed in cancerous cells treated with the tested *N.retusa *leaf extracts. As far as the extracts tested in the present study were in crude form and probably contained many compounds which may well act synergistically. It is not possible to say which compounds are responsible for the observed effects. However, our data suggest that the biological effects exhibited by this plant, under these experimental conditions, could be related to an overall effect of the tannins, flavonoids, sterols and coumarins present in these extracts. Phenolic compounds were postulated as effective in inducing apoptosis and as anticancer agents [39].

Likewise steroids showed growth inhibition of human prostate cancer PC-3 cells, being effective in inducing apoptosis [40].

As far as we obtained at all tested concentrations of different extracts, the activation of caspases 3 and 8, as well as the more effective PARP cleavage effect, after 48 h of incubation, we can deduce that the tested extracts should provoke a cytotoxic effect towards K562 cells by activating the extrinsic pathway of apoptosis. In fact, activation of caspase 8 leads to the activation of caspase 3 and subsequently induces PARP cleavage (the 116 kDa band disappears in favour of 85 kDa band) and DNA fragmentation (ladder electrophoretic profile). However we can not exclude the participation of other pathways in the apoptotic effect exhibited by these extracts.

## Conclusion

In summary, *N.retusa *leaf extracts appear to contain compounds with, antiproliferative and apoptotic properties. The three tested extracts induced apoptotic effect by the activation of the extrinsic apoptotic pathway. As apoptosis has become a new therapeutic target in cancer research, it appears reasonable to suggest that *N.retusa *may have potential as an agent of chemotherapeutic and cytostatic activity in human leukaemia lymphoma.

## Competing interests

The authors declare that they have no competing interests.

## Authors' contributions

BJ:Was responsible for the conception and design, testing and data acquisition, analysis and data interpretation and drafted the manuscript. BW:made contribution to the study of caspase activities. BSG:made contribution to cell culture and the study of the DNA fragmentation.

IB:made contribution to cell culture and the study of the cytotoxicity. SI:made contribution to data interpretation and drafted the manuscript. GK: made substantial contribution to conception and revised it critically for importantintellectual content. CGL:made substantial contribution to conception and revised it critically for important intellectual content.

All authors read and approved the final manuscript.
